# Karyotype and nuclear DNA content of hexa-, octo-, and duodecaploid lines of *Bromus* subgen. *Ceratochloa*

**DOI:** 10.1590/S1415-47572009005000046

**Published:** 2009-09-01

**Authors:** Joanna Klos, Elwira Sliwinska, Adam Kula, Hieronim Golczyk, Aleksandra Grabowska-Joachimiak, Tomasz Ilnicki, Krzysztof Szostek, Alan Stewart, Andrzej J. Joachimiak

**Affiliations:** Cytogenetics Group in the Department of Plant Breeding and Seed Science, University of Agriculture in Kraków, CracowPoland; 2 Laboratory of Molecular Biology and Cytometry, Department of Genetics and Plant Breeding, University of Technology and Life Sciences, BydgoszczPoland; 3 Department of Plant Cytology and Embryology, Institute of Botany, Jagiellonian University, CracowPoland; 4 Department of Anthropology, Institute of Zoology, Jagiellonian University, CracowPoland; 5 PGG Wrightson Seeds, ChristchurchNew Zealand

**Keywords:** karyotype, C-banding, heterochromatin, flow cytometry, genome size

## Abstract

The subgenus *Ceratochloa* of the genus *Bromus* includes a number of closely related allopolyploid forms or species that present a difficult taxonomic problem. The present work combines data concerning chromosome length, heterochromatin distribution and nuclear genome size of different 6x, 8x and 12x accessions in this subgenus. Special attention is paid to the karyotype structure and genomic constitution of duodecaploid plants recently found in South America. Hexaploid lineages possess six almost indistinguishable genomes and a nuclear DNA content between 12.72 pg and 15.10 pg (mean 1Cx value = 2.32 pg), whereas octoploid lineages contain the same six genomes (AABBCC) plus two that are characterized by longer chromosomes and a greater DNA content (1Cx = 4.47 pg). Two duodecaploid accessions found in South America resemble each other and apparently differ from the North American duodecaploid *B. arizonicus* as regards chromosome size and nuclear DNA content (40.00 and 40.50 pg *vs.* 27.59 pg). These observations suggest that the South American duodecaploids represent a separate evolutionary lineage of the *B*. subgenus *Ceratochloa*, unrecognized heretofore.

## Introduction

The genus *Bromus* resembles the majority of festucoid grasses in chromosome size (medium to large) and basic chromosome number (x = 7). Distinct groups (sections or subgenera) of *Bromus* are distinguished mainly on the basis of morphology, ploidy level, serological or genomic relationships and karyotype structure (Smith, 1972; Stebbins, 1981; Armstrong, 1983, 1984, 1991; Joachimiak *et al.*, 2001). Polyploidy is widespread in *Bromus* (Stebbins 1981), and within three subgenera (*Festucaria*, *Bromus*, *Stenobromus*) both diploids and polyploids have been recognized. No diploid species has been reported in the American subgenera *Ceratochloa* and *Neobromus*, the lowest reported ploidy level being 6x (Stebbins, 1981). Currently, no species of these subgenera is regarded as being indigenous to the Old World.

The evolutionary history of the genus *Bromus*, and especially of the American subgenera *Ceratochloa* and *Neobromus,* has been outlined by Stebbins (1981). He suggested that the whole genus originated in Eurasia, and that many of its representatives underwent further evolutionary changes when migrating to the Americas. According to this hypothesis, Eurasia was also the center of differentiation of the diploid, tetraploid, and most probably, the hexaploid species that became the ancestors of the two American subgenera. During the Pliocene Era, some of these species migrated to the Americas. Further climatic changes and competition from modern species belonging to other *Bromus* groups contributed to the extinction of the Eurasian ancestors of the American forms. This explains why these subgenera no longer include any diploid and tetraploid species. Contemporary analyses of nuclear and chloroplast DNA (Pillay and Hilu, 1995; Saarela *et al.*, 2006, 2007) confirmed that the two subgenera are more closely related to each other than to the subgenera *Festucaria*, *Stenobromus* or *Bromus*.

A further evolution of the subgenus *Ceratochloa* occurred in the New World during the Pleistocene, this resulting in the differentiation of the modern South American hexaploid species, all closely related to each other (*B. catharticus* complex), and the formation of higher allopolyploids (8x, 12x) of subgeneric origin in North America (Stebbins *et al.*, 1944; Stebbins, 1947). No hexaploid species belonging to this subgenus and native to North America occurs nowadays. Thus, the ancestral hexaploids were most probably eliminated from this continent by the superior competitive ability of their octoploid (*B. carinatus* complex) and duodecaploid (*B. arizonicus*) derivatives (Stebbins, 1981).

The evolutionary picture outlined by Stebbins is well established, and until now, almost all morphological, cytological and molecular data are consistent therewith. Nevertheless, some questions remain unsolved, especially with respect to the existence of higher than 6x polyploids native to South America. In his work, Stebbins (1981) stressed that almost all species of the subgenus *Ceratochloa*, with the sole exception of *B. arizonicus*, a duodecaploid with 84 chromosomes, can be divided into two series, the 6x - *B. catharticus* complex and the 8x - *B. carinatus* complex, on the basis of their genomic constitution, which runs in parallel with certain morphological characteristics, and geographical distribution. All 6x species are allohexaploids, contain the same three medium-sized genomes (designated A, B and C by Stebbins) and are indigenous to South America. All octoploid species also possess very similar karyotypes, with 42 medium-sized chromosomes (homologous with the *B. catharticus* chromosome complex) and 14 large ones (two L complexes, derived from unknown species of subgen. *Festucaria*), thereby indicating a common, or at least very similar, origin.

According to most authors, the South American subgenus *Ceratochloa* is both restricted to hexaploid species and separate from the octoploid North American *Ceratochloa* species (Massa *et al.*, 2004). Nevertheless, the occurrence of native octoploid *Ceratochloa* populations on the southern subcontinent has been suggested by Stebbins (1947, 1981) (octoploid *B. pittensis*) and supported by Massa *et al.* (2001) (two different accessions from Chile and Argentina). Furthermore, Stebbins (1981) has also proposed that all the North American octoploids should be united into a single species, *B. carinatus*, due to their presumed common origin, and that the South American octoploid species, unlike its North American relatives, may be of independent origin, having obtained two larger genomes from a species of the *Bromus* subgenus *Festucaria*.

The evolutionary history of the South American octoploids as belonging to the subgenus *Ceratochloa* is very poorly understood. Two South American accessions analyzed by Massa *et al.* (2001) and classified through morphology as *B. coloratus* and *B. lithobius*, are cytogenetically indistinguishable from the North American octoploids. AFLP studies have confirmed the supposition that all American octoploids share a common set of 21 chromosome pairs (*i.e.*, AABBCC genomes), and possess additional *Festucaria* genomes, absent from *Ceratochloa* hexaploid accessions. There are, however, no notable results documenting the independent origin of the two larger genomes in South and North American octoploids. Thus, it is still possible that all octoploid representatives of the subgenus *Ceratochloa* share the same genome and could be regarded as a single, though highly polymorphic, species.

The merging of different *Ceratochloa* populations/species that share the same chromosome number and the same genomic composition into one single collective species is a reasonable proposition, at least from the evolutionary point of view. Stebbins (1981) suggested that within distinct lineages it is extremely difficult to delimit taxa on the basis of either external morphology or the degree of reproductive isolation. The most recent morphological and molecular studies by Massa *et al.* (2001, 2004) on South American *Ceratochloa* accessions support this view, and confirm the impracticability of clearly delimiting taxa within this group, as the patterns of genetic variation within and among the different populations analyzed were in disagreement with all previous morphological classifications, only two South American species having been distinguished, namely *B. catharticus* (6x) and *B. coloratus* (8x), these differing mainly at the ploidy level.

The North American duodecaploid *B. arizonicus*, described and recognized as a distinct species by Stebbins *et al.* (1944), constitutes the third evolutionary lineage within the subgenus *Ceratochloa*. In contrast to all American octoploids, this highly polyploid species contains only medium-sized chromosomes and is completely incapable of interbreeding. Morphological and cytological analyses by Stebbins *et al.* (1944) indicate that *B. arizonicus* is most probably a quite different intersubgeneric allopolyploid derived from *B. catharticus* and the hexaploid *B. trinii*, or an unknown close relative to the latter. *B. trinii* is a representative of the subgenus *Neobromus*, native to the Pacific coast of North and South America.

The aim of this study was to characterize the nuclear genomes of the hexaploid, octoploid and duodecaploid lines of *Bromus*, subgenus *Ceratochloa*, by means of chromosome size, Giemsa C-banding and nuclear DNA content, to then use the obtained information to examine the relationship of these lines, with special reference to the genomic composition of two newly discovered South American duodecaploid forms of this subgenus. In this way the evolutionary relationships of different lineages could be determined.

## Material and Methods

###  Plant material

Seeds from 20 different hexa-, octo- and duodecaploid accessions, 3 widely cultivated hexaploids and 1 cultivated octoploid, were analyzed (Table 1). Nineteen accessions represented the subgenus *Ceratochloa*, whereas one (hexaploid *B. trinii)* was a close relative belonging to the subgenus *Neobromus*.

###  Cytology

Seeds were germinated on moistened blotting paper in Petri dishes. Root tips from 3 or 4 days-old seedlings were pre-treated with a saturated aqueous solution of α-bromonaphthalene for 2-4 h, fixed in ethanol/glacial acetic acid (3:1) and then stored in a refrigerator. For conventional chromosome analysis, root tips were stained with acetic orcein. Squashes were made in 45% (v/v) acetic acid. Chromosome counts for each accession were carried out from 3-6 complete metaphase plates obtained from 3 different seedlings. For chromosome length measurements, metaphase plates were selected from 9 hexaploid (CT1-CT8, NB), 4 octoploid (CT17-CT20), and 3 duodecaploid (CT21-CT23) accessions (Table 2).

Squash preparations derived according to Grabowska-Joachimiak and Joachimiak (2002) and stained by a slightly modified version of the C-banding method of Jouve *et al.*, (1980) were used in the study of C-banding chromosomes. Briefly, the cover slips were removed from frozen preparations which were subsequently air-dried, incubated in absolute ethanol for about 24 h, and then in 0.2 M HCl for 2 min at 60 °C, rinsed under tap water and in distilled water, incubated in a 3% (w/v) Ba(OH)_2_ solution for 5 min at 38 °C, rinsed under warm tap water until completely clear, incubated in 2 x SSC buffer for 1 h at 60 °C, and stained in 2% (w/v) Giemsa solution (in Sorensen buffer, pH 6.9) for about 45 min. The overall C-banding style of the chromosomes (the C-band position therein) was assessed for 17 accessions and at least 2 chromosome preparations. In the case of accessions CT9, CT12, CT14, CT15, CT20 and NB (Table 1), C-banding analysis failed either through the lack of satisfactorily stained preparations, chromosome fragmentation, or difficulties in obtaining enough viable seedlings. The amount of heterochromatin measured for 8 hexaploid accessions (CT1-CT8) was calculated as a percentage of C-banded karyotype length. For each accession, three complete metaphase plates showing the maximum banding response were selected for analysis.

Chromosome images were captured and processed by using a CCD camera and LUCIA G software (Laboratory Imaging Ltd., Praha, Czech Republic). The complete, well-spread metaphase plates of duodecaploid plants were large, and thus usually captured in 2-4 overlapping fragments.

###  Flow cytometry

For flow cytometric analysis, samples were prepared as previously described (Grabowska-Joachimiak *et al.*, 2006). *Pisum sativum* cv. Set (2C = 9.11 pg/nucleus; Sliwinska *et al.*, 2005) was used as internal standard. A buffer consisting 0.1 M Tris, 2.5 mM MgCl_2 ._6H_2_O, 85 mM NaCl and 0.1% (v/v) Triton X-100, supplemented with propidium iodide (50 μg/mL) and ribonuclease A (50 μg/mL), was used to isolate nuclei. For each sample, 8,000-10,000 nuclei were analyzed by means of a Partec CCA flow cytometer (Münster, Germany) equipped with an argon laser. Ten measurements of separate nuclei isolations from different plants were taken for each accession. Histograms were analyzed with DPAC v.2.2 software. Nuclear DNA content was calculated by using the linear relationship between the ratio of the 2C *Bromus*/*Pisum* peak positions on the histogram of fluorescence intensities.

###  Statistical analysis

Correlation analyses and other statistical studies were undertaken using STATGRAPHICS Plus software, version 5.0 (StatPoint, Inc., USA). Linear regression analysis was applied to examine the dependence between 2C DNA value and heterochromatin amount. An outlier plot with sigma limits was employed for classifying chromosomes in relation to length. Eleven chromosome classes were distinguished by cross-tabulation analysis. All classes with the exception of the medium one were created through 0.5 standard deviation steps. In the construction of a mosaic chart, chromosomes were arbitrarily divided into medium (1-6 μm) and long (7-11 μm) classes.

## Results

Of the 23 *Bromus* subgenus *Ceratochloa* accessions analyzed, 16 showed 2n = 6x = 42 chromosomes, four 2n = 8x = 56 chromosomes and three 2n = 12x = 84 chromosomes (Table 1). For flow cytometric DNA-histograms, the mean CV-value of the G_0_/g_1_ peak of a sample was 4.74% and of an internal standard, 5.03%.

###  Hexaploids

**Figure 1:**
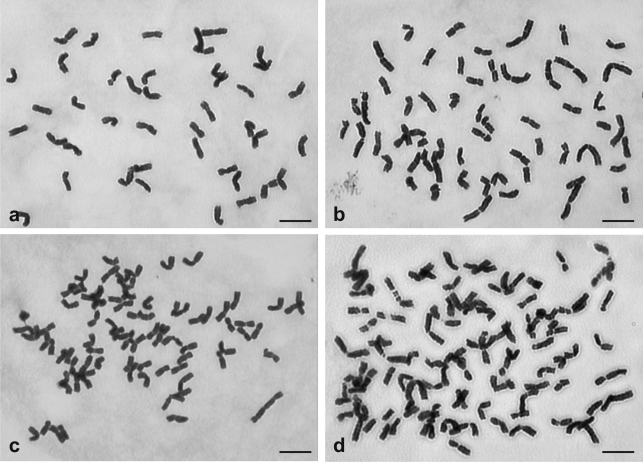
Metaphase chromosomes in four different American lines of *Bromus* subgen. *Ceratochloa*. a - hexaploid *B. willdenowii* cv. *Atom* [CT3], b - octoploid *B. marginatus* [CT20], c -duodecaploid *B. arizonicus* [CT21], d - duodecaploid from Machachi (Ecuador) [CT22]. Note the presence of longer chromosomes in CT20 and CT 22. [] indicates origin of *Bromus* samples (see Table 1). Scale bar = 5 μm.

All of the 9 karyotyped lines (8 spp. of *B*. subgenus *Ceratochloa* and 1 sp. of *B*. subgenus *Neobromus*) possessed similar chromosome sets composed of poorly distinguishable medium-sized chromosomes. Within particular metaphases, differences in length between chromosomes were small (Figure 1a). However, the analyzed metaphase plates were differently condensed. Thus, chromosome lengths ranged from 2.14-7.12 μm within the whole chromosome collection (Table 2). After normalization (chromosome length expressed as a % of the karyotype), all forms showed a similar chromosomal distribution.

**Figure 2:**
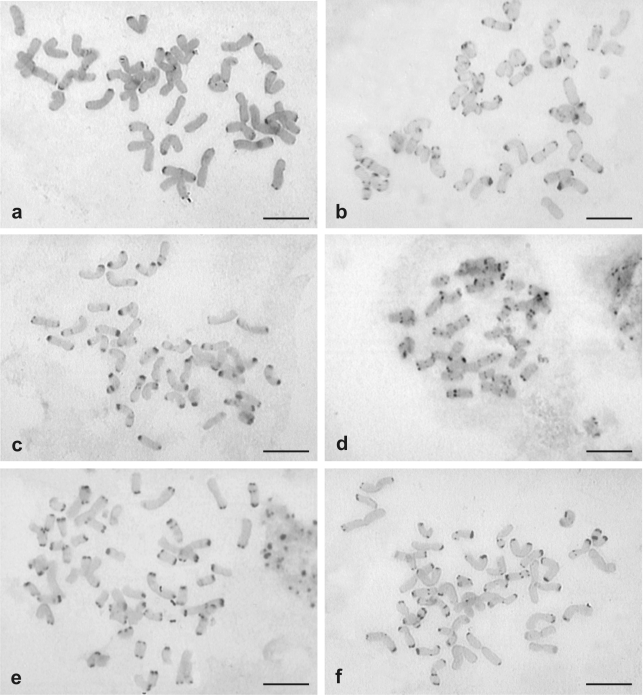
C-banded chromosomes of different hexaploid (a-d), octoploid (e) and duodecaploid (f) lines of *Bromus* subgen. *Ceratochloa*. a - [CT3], b - [CT5], c - [CT8], d - [CT10], e - [CT17], f - [CT22] (metaphase fragment). [] indicates the origin of *Bromus* samples (see Table 1). Scale bar = 5 μm.

C-bands also revealed a rather uniform distribution within a particular karyotype: the majority of the chromosomes were equipped with distally located heterochromatin (Figure 2a-c). However, different lines differed in the amount of heterochromatin within each karyotype: from 4.34% in *B. willdenowii* cv. Atom (CT3) to 9.59% in *B. russorensis* (CT8) (Table 2). Although telomeric distribution of heterochromatin seems to be usual in this group, two hexaploid accessions not analyzed here in detail (CT10 from Argentina, CT13 from Mexico) showed a number of additional, interstitially-located C-bands (Table 1, Figure 2d). Interestingly, both accessions showed a substantial reduction in the size of terminally located C-bands.

**Figure 3:**
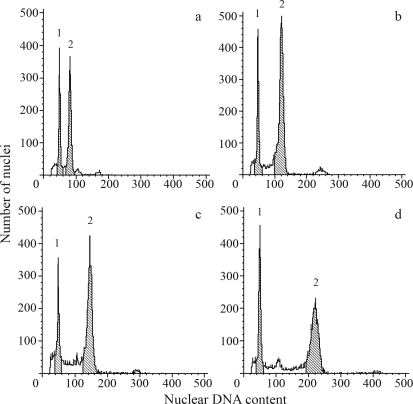
Histograms of fluorescence intensities of nuclei isolated from the leaves of different lines of *Bromus* subgen. *Ceratochloa* (peak 2) stained simultaneously with the nuclei of *Pisum sativum* (internal standard; peak 1) by using propidium iodide. a - hexaploid [CT8], b - octoploid [CT19], c - North American duodecaploid [CT21], d - South American duodecaploid [CT23]. [] indicates origin of *Bromus* samples (see Table 1).

The nuclear 2C DNA content of eight hexaploid *Ceratochloa* accessions ranged from 12.72 pg to 15.10 pg (mean value = 13.91 pg) (Table 2, Figure 3a). The average DNA content per monoploid genome (1Cx) of these lines was 2.32 pg, with a variation of 2.12 to 2.82 pg. *B. trinii* (subgenus *Neobromus*), another American hexaploid, contained a similar chromosome set, although its nuclear DNA content was slightly lower than in most of the other hexaploid *Bromus* ssp. (1Cx = 2.09; Table 2).

**Figure 4:**
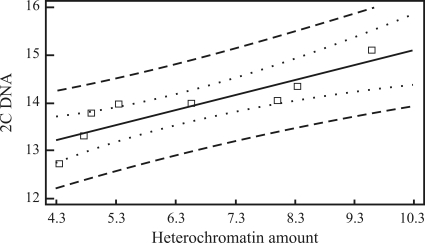
Regression of 2C DNA value (in pg) on heterochromatin amount (in % of karyotype length) in eight hexaploid species; linear model represents statistical dependence between the analysed variables (p = 0.043, r = 0.877, r^2^ = 79.6%).

The observed differences in nuclear DNA content of *B*. subgenus *Ceratochloa* hexaploids might be a result of variation in the amount of heterochromatin within the karyotype, for the accession with the highest 2C DNA content (CT8; Figure 2c) also showed the highest amount of heterochromatin, whereas the accession with the lowest 2C DNA content (CT3; Figure 2a) the lowest. Regression analysis revealed a significant relationship between 2C DNA value and the amount of heterochromatin at the 99% confidence level (correlation coefficient = 0.877). The R-squared statistic indicates that the linear model (2C DNA = 11.8802 + 0.313421 x heterochromatin amount) provides explanations for the 79.6% variability in the amount of 2C DNA in the analyzed plants (Figure 4).

###  Octoploids

Chromosome lengths in octoploids ranged from 2.07-8.74 μm (Table 3). The difference in karyotype length among hexa- and octoploid forms most probably resulted from the presence of two longer (L) genomes within the karyotype itself (genome formula suggested by Stebbins: AABBCCLL). Fourteen long chromosomes belonging to these genomes were easily identified in some metaphase plates (Figure 1b). In strongly condensed metaphases, however, identification of all those chromosomes belonging to L genomes was difficult, as all the plants showed a similar, telomeric heterochromatin distribution (Figure 2e). Furthermore, there was no detectable difference in C-band distribution between medium-sized and long chromosomes.

The amount of nuclear DNA of three analyzed 8x accessions ranged from 2C = 22.66 pg to 2C = 22.97 pg (mean value 22.86 pg) (Table 3, Figure 3b). The size of two additional L genomes in all these forms was close to 9 pg. Thus, the 1Cx value calculated for a single L genome (~4.5 pg) was considerably greater than that estimated for the basal genome of hexaploids (1Cx = 2.32 pg, Table 2).

###  Duodecaploids

**Figure 5:**
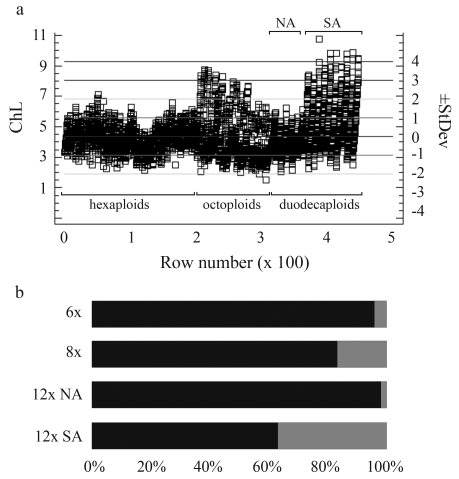
Chromosome size distribution in chromosome collections of hexa-, octo- and duodecaploid *Bromus* subgenus *Ceratochloa*. a - figure shows summary statistics for central tendency and variability of chromosome lengths, and displays the usual estimates of means and standard deviation, b - mosaic chart showing the frequency of medium-sized (black) and long (grey) chromosomes in four different lineages. ChL - chromosome length (μm), StDev - standard deviation, Row number - number of analyzed chromosomes (cumulatively), NA - North American duodecaploid [CT21], SA - South American duodecaploids [CT22, CT23]. Sample mean = 4.38, std. deviation = 1.23.

All chromosomes of the North American *B. arizonicus* were medium-sized (Table 3, Figure 1c), their lengths (2.07-6.24 μm) being within the size-range of the analyzed hexaploid forms (Table 2). Chromosome-size distribution (Figure 5a, b) strongly confirmed the outstanding similarity of the nuclear genomes of *B. arizonicus* to those of the hexaploid forms. The nuclear DNA value for *B. arizonicus* (2C = 27.59 pg, Table 3, Figure 3c) was nearly twice that of the hexaploids (calculated size of six additional genomes = 13.68 pg).

Two South-American 12x accessions greatly differed from *B. arizonicus* in respect of both chromosome size and nuclear DNA amount, although they were very similar to each other (Table 3, Figures 3d and 5a). Besides medium-sized chromosomes, they contained many large ones (Figs. 1d), some being even longer than the longest chromosomes in the L genome of North American octoploids (Table 3, Figure 5a). There was also a significant difference between the contribution of large-sized chromosomes in the karyotypes of these duodecaploids (36.9%) and that in the North American octoploids (17.33%) (Figure 5b). Nevertheless, all manifested telomeric heterochromatin distribution (Figure 2f).

The assumption of the difference in genome composition between the North American *B. arizonicus* and the two South American duodecaploids was supported by 2C DNA estimates (Table 3, Figure 3d). The evolutionary origin of South American duodecaploids is unknown, but it can be speculated that, as is the case of other *B*. subgen. *Ceratochloa* lines, they possess the basal hexaploid (AABBCC) complement of medium-sized chromosomes. Using this supposition, the size of the six additional genomes comes to about 26 pg (Table 3). Thus, the mean size of a single additional genome is 4.39 pg, which is a very similar value to that of the L genome in the octoploid *B*. subgen. *Ceratochloa* (4.47 pg).

## Discussion

One feature present in all hexa- and octoploid karyotypes, and most probably common within the subgenus *Ceratochloa*, is the occurrence of a uniform set of 42 medium-sized chromosomes. Unfortunately, three genomes of this set, designated by Stebbins (1981) as A, B and C, are very similar. They contain a large amount of telomeric heterochromatin, present in almost all the *Bromus* genomes analyzed by C-banding (Armstrong, 1991; Kula, 1999; Joachimiak *et al.*, 2001; Tuna *et al.*, 2001, 2004, 2006). Despite the uniform AFLP profile (Massa *et al.*, 2001), the basal 42-chromosome set of different hexaploid accessions shows detectable differences, both in DNA amount and the size of distal heterochromatin segments, thereby suggesting that minor chromosomal changes occurred during the evolution of the different 6x lineages, most probably, through the gain or loss of highly repeated sequences. On the assumption that the ancestral *B. catharticus* s.l. (2n = 42) karyotype was characterized by terminally-located heterochromatin, the karyotypes of accessions CT10 and CT13 (Table 1), through showing a large number of interstitially-located bands and a reduction of terminally-located heterochromatin, seem to be the most evolutionarily advanced. A number of similarly distributed C-bands have been observed previously only in the karyotype of the tetraploid *B. ciliatus*, the North American species of the subgenus *Festucaria* (Tuna *et al.*, 2005).

In spite of certain differences in nuclear DNA content between the eight hexaploid accessions analyzed herein, the mean 2C DNA value (13.91 pg) is very similar to that calculated from *B*. subgenus *Ceratochloa* records in the RGB Kew Plant DNA C-value Database (Bennett and Leitch, 2005; 14.26 pg). The DNA content of one monoploid genome calculated from these two data sets stands at about 2.3 pg. Hexaploid *B. trinii*, the only representative of the other American subgenus *Neobromus*, presents a very similar chromosome set, but a slightly lower nuclear 2C DNA content. This is not quite in agreement with the results arrived at by Pillay and Hilu (1995) and Saarela *et al.* (2006), who observed a similarity between the subgenera *Neobromus* and *Ceratochloa* at the DNA level. These authors also demonstrated a clear difference between the Eurasian and American *Bromus* lineages, but a very close relationship between the two American subgenera.

Two additional L genomes occurring in octoploids are longer and possess more DNA (~4.5 pg per genome). Stebbins (1981) suggested that these two genomes originated from the subgenus *Festucaria,* and this was confirmed by Pillay (1996), who found certain rDNA variants common to *B. inermis* (subgenus *Festucaria*) and North American octoploids. The octoploid accessions analyzed here appear to be karyologically uniform, since they are very similar as regards chromosome length, 2C DNA content and heterochromatin amount and distribution. All these observations suggest a single ancestry and the evolutionary stability of the chromosome set of North American octoploids. In contrast, hexaploid forms, although similar to each other, are more variable in genome size and C-band distribution.

Chromosome and 2C DNA analysis revealed considerable differences between the North American and South American duodecaploids. The lineage of the former (*B. arizonicus*) shows only medium-sized chromosomes, their length distribution being very similar to that in hexaploid accessions (Figure 5). The size of six additional genomes of this species was calculated at 13.68 pg. Thus, the size of a single additional genome was 2.28 pg, which is very similar to the average size of a single genome in the hexaploids *B*. subgenus *Ceratochloa* (1Cx = 2.32 pg) and *B. trinii* (1Cx = 2.09 pg). In contrast, two South American duodecaploids manifested more differentiated karyotypes with both medium-sized and long chromosomes. The size of the six additional genomes of the South American accessions was nearly 26 pg. The calculated size of a single additional genome (4.39 pg) was almost the same as that of the L genome in North American octoploids (4.47 pg). All these observations suggest a very different origin for the North American and South American duodecaploids.

Two analyzed South American lines, one collected at higher altitudes in Machachi, Ecuador (3000 m a.s.l.), and the other about 50 km south of Bogota, in Colombia (2600 m a.s.l.), are different not only as regards karyology but also morphology. They show lanceolate, strongly compressed spikelets, strongly keeled lemmas and glumes (characteristic of the subgenus *Ceratochloa*), 1-veined lower glumes and 3-veined upper ones (unusual characters in the subgenus *Ceratochloa*, where the lower glumes are 3-7(9)-veined and the upper ones 5-9- veined; Smith 1970). This combination of characters had been previously described only for *B. ayacuchensis*, a new species of *B*. subgenus *Ceratochloa*, recently found in Peru (3730 m a.s.l.) (Saarela *et al.*, 2006). It is possible that all these forms represent the same lineage, although the accessions analyzed here showed different-shaped ligulae.

In summary, the combined use of cytogenetic analysis and nuclear DNA content demonstrates karyotypical uniformity in the hexaploid (AABBCC) and octoploid (AABBCCLL) accessions of *Bromus* subgenus *Ceratochloa*. The differences in nuclear DNA amount between lineages at the same ploidy level are small, and most probably result from the gain or loss of heterochromatic sequences. In contrast, different duodecaploid forms of *B*. subgenus *Ceratochloa* are more variable as to nuclear DNA content and genomic composition. The accessions from South America are mutually similar, although very different from the North American *B. arizonicus.* Most probably they represent the fourth evolutionary lineage within the subgenus *Ceratochloa*, native to South America and characterized by a distinct genomic combination.

## Figures and Tables

**Table 1:** Provenance, chromosome number (ChN) and C-band distribution (CBD) of *Bromus* accessions.

	Place (access. number/cultivar)	Species	ChN	CBD
CT1	PI 202014, Argentina^a^	*B. brevis*	2n = 42	t
CT2	BARENO^cv^, germplasm collected in Chile	*B. valdivianus*	2n = 42	t
CT3	ATOM^cv^, germplasm collected in NZ	*B. willdenowii*	2n = 42	t
CT4	GALA^cv^, germplasm collected in Chile	*B. stamineus*	2n = 42	t
CT5	PI 409138, Roseberg Pass, South Africa^a^	*B. leptoclados*	2n = 42*	t
CT6	Blue Mountains, Australia^b^	*B. stamineus*	2n = 42	t
CT7	Christchurch, New Zealand^b^	*B. lithobius*	2n = 42	t
CT8	Cb1061, Ethiopia^b^	*B. russorensis*	2n = 42^**^	t
CT9	Tas535, Chile^b^	*B. mango*	2n = 42	*ne*
CT10	RGA30, Santa Cruz, Argentina^b^	*B. unioloides*	2n = 42	t/nt
CT11	Mar del Plata, Argentina^b^	*B. bonarensis*	2n = 42	t
CT12	PI 306289, Bolivia^b^	*B. unioloides*	2n = 42	*ne*
CT13	Morelos, Zacatecas, Mexico^b^	*B. unioloides*	2n = 42	t/nt
CT14	IAT509, McLean, Texas, USA^b^	*B. stamineus*	2n = 42	*ne*
CT15	Tulcan, Ecuador^b^	*B. unioloides*	2n = 42	*ne*
CT16	PI 308506, Peru^b^	*B. unioloides*	2n = 42	t
NB	Botanic garden origin	*B. trinii*	2n = 42	*ne*

CT17	#15, Oregon Coast, USA^c^	*B. carinatus*	2n = 56	t
CT18	BROMA^cv^ (J)	*B. carinatus*	2n = 56***	t
CT19	#16, Fort Bragg, Oregon, USA^c^	*B. maritimus*	2n = 56	t
CT20	PI 236755, British Columbia, Canada^a^	*B. marginatus*	2n = 56	*ne*

CT21	PI 469231, Cucamonga ^cv^, USA^a^ (*carinatus*)	*B. arizonicus*	2n = 84	t
CT22	Ecuador, Machachi, south of Quito^b^	undetermined	2n = 84	t
CT23	Colombia, Bernardo, south of Bogota^b^	undetermined	2n = 84	t

CT: subgen. *Ceratochloa*, NB - subgen. *Neobromus* ChN: chromosome number; CBD - C-band distribution: t - majority of heterochromatin located terminally, t/nt - karyotypes with a considerable amount of non-terminally located heterochromatin, *ne* - not examined ^cv^: commercial variety; ^a^: USDA genebank; ^b^: Alan Stewart (Christchurch, New Zealand) collection; no accession number; ^c^: David Amme, California, USA collection. (J): ChN and CBD determined by Joachimiak *et al.*, 2001; (*carinatus*) - commercial duodecaploid line sold under the name *B. carinatus*. *: also 56, 65, 74, 82. **: also 28, 35. ***: chromosome number in root-tip cells highly unstable (Joachimiak *et al.*, 2001).

**Table 2:** Chromosome length, heterochromatin amount, and nuclear DNA content in hexaploid *Bromus* subgen. *Ceratochloa* (*B. catharticus* sp. coll.) and *B. trinii* accessions. 1Cx - DNA content of one non-replicated monoploid genome with chromosome number x (according to Greilhuber *et al.*, 2005).

Accession/species	Chromosome length min-max (μm)	Total length of karyotype (μm, mean ± SD)	Length of basal chromosome set (x) (μm)	Amount of heterochromatin (% of karyotype length)	2C (pg, mean ± SD)	1Cx (pg)
[CT1] *B. brevis*	2.23-5.93	172.13 ± 12.72	28.69	4.76 ± 0.37	13.31 ± 0.20	2.22
[CT2] *B. valvidianus*	3.05-6.20	184.85 ± 12.03	30.81	8.34 ± 0.68	14.35 ± 0.22	2.39
[CT3] *B. willdenowii*	2.72-6.30	182.74 ± 8.28	30.46	4.34 ± 0.51	12.72 ± 0.08	2.12
[CT4] *B. stamineus*	2.55-6.30	171.39 ± 17.80	28.57	4.88 ± 0.76	13.79 ± 0.12	2.30
[CT5] *B. leptoclados*	2.14-4.70	144.83 ± 7.53	24.14	6.56 ± 1.74	13.99 ± 0.19	2.33
[CT6] *B. stamineus*	2.73-7.12	195.60 ± 15.26	32.60	8.00 ± 0.58	14.04 ± 0.15	2.34
[CT7] *B. lithobius*	2.52-5.89	188.99 ± 7.29	28.16	5.34 ± 0.31	13.98 ± 0.18	2.33
[CT8] *B. russorensis*	3.37-6.15	191.10 ± 2.67	31.85	9.59 ± 1.89	15.10 ± 0.22	2.52
	mean ± SD	178.95 ± 16.22	29.41 ± 2.67	6.48 ± 1.96	13.91 ± 0.70	2.32 ± 0.12

[NB] *B. trinii*	2.90-6.56	186.80 ± 18.27	31.13	(-)	12.52 ± 0.19	2.09

**Table 3:** Chromosome length and nuclear DNA content in octoploid and duodecaploid accessions. AD - amount of DNA in two additional (octoploid) and six additional (duodecaploid) genomes, calculated as measured 2C DNA amount minus average 2C DNA amount of hexaploid *Bromus* subgen. *Ceratochloa* (*B. catharticus* sp. coll.) complement.

Accession/species	Chromosome length min-max (μm)	Total length of karyotype (t) (μm, mean ± SD)	2C (pg, mean ± SD)	AD (pg)
[CT17] *B. carinatus*	2.47-8.37	259.64 ± 12.20	(-)	(-)
[CT18] *B. carinatus*	2.07-8.74	254.68 ± 23.27	22.94 ± 0.42*	9.03
[CT19] *B. maritimus*	2.09-7.84	231.24 ± 25.38	22.97 ± 0.24	9.06
[CT20] *B. marginatus*	2.25-7.96	238.85 ± 21.97	22.66 ± 0.56	8.75
	Mean ± SD	246.10 ± 13.30	22.86 ± 0.17	8.95 ± 0.17

[CT21] *B. arizonicus*	2.07-6.24	333.97 ± 21.54	27.59 ± 0.22	13.68
[CT22] undetermined	2.25-10.74	436.13 ± 8.67	40.50 ± 0.73	26.59
[CT23] undetermined	2.36-9.87	451.01 ± 29.76	40.00 ± 0.77	26.09

*According to Joachimiak *et al.*, 2001.
